# The Medical Bill

**Published:** 1891-02

**Authors:** 


### THE MEDICAL BILL.

The bill to regulate the practice of medicine in Texas, prepared by the committee on legislation of the Texas State Medical Association, and now before the Senate, provides for a Board of Medical Examiners of nine, of whom one shall be a homeopath, and one an eclectic. The board is to meet at Austin, semi-annually, and hereafter all persons essaying to practice medicine or surgery,—diploma or no diploma,—are to be examined. Thus, it will be seen, that it does not interfere with any one now practicing who has complied with any law on the subject, now or heretofore in existence.

When the House Committee on Public Health met, early in January, and considered the "Pope bill," and others, (before this bill had been introduced,) a homeopathic representation presented themselves, armed with the usual protest, and on the Pope bill being read, they fired into it, under the impression that it was the bill from the Texas State Medical Association. In their protest they said, "The administration of our remedies is harmless,—why can they not let us alone—leave us out?" The Association's committee took them at their word, and introduced a clause which exempts from examination all who practice homeopathy exclusively, *i. e.*, adheres to *similia similabus*, and infinitesimal doses. This should disarm all opposition, as they have asked for it; but it is a trap into which they have unwittingly fallen, for, as is well known, the great majority of those who call themselves homeopaths, do not hesitate to practice, or attempt to practice, what they call "allopathy," when their "little truck" makes no impression—*i. e.*, in grave cases; and nearly or quite all of them use the hypodermic syringe. Hence, should the bill pass, it would seem that these gentlemen would be confined to their legitimate sphere, the practice of a harmless "pathy," and when they deviate from it, would be amenable to the law. But how about surgery and obstetrics? We cannot comprehend the idea of homeopathic surgery; yet they have colleges in which surgery is taught, and they all essay to practice it. It does seem to us that, claiming, as they do, the equality of their colleges with any in the land, and surgery and obstetric being taught alike in all, they should be exempted from examination only on practice, and that there would be no need of a representative on the board. We doubt the wisdom of the step taken.

There are less than one hundred homeopaths in Texas, all told, not enough to make a fuss about, yet they have the assurance to ask for equal representation in all government medical affairs.

The title of the bill is unfortunate. It is not a bill to regulate the practice, but to prohibit the indiscriminate practic of medicine, or, to restrict it to the hands of those who can give evidence of qualification for the privilege; a bill, in short, to prevent quackery. The word "regulate" carries with it the idea of "control" by the State, or by the socalled "allopaths," and no doubt has caused much of the opposition from the other so-called "schools;"—for they are, or pretend to be, as much opposed to quackery as any one. Were the title changed, as suggested, there would seem to be no grounds of opposition.

In the house the bill will have the support of a number of sensible and liberal minded physician-members, and there are strong grounds to hope for its passage in some form that will compas the end sought—prevent the ignorant from tampering with the lives and health of the people.

Numerous other bills have been introduced,—so many, in fact, that we cannot even give a synopsis of them. Dr. Tally's bill, creating a State Board of Health, and making the State Health Officer president of it, is now before the Senate. The bill abolishes the office of State Health Officer. Dr. Cunningham's bill calls for a Board of Health in each county, and defines its duties. The T. S. M. A. Committee have introduced no bill for a Board of Health, nor has the special committee to procure a Board of Health,—appointed at the Fort Worth meeting last April, upon the recommendation of retiring President Swearingen,—been heard from. The President, Dr. Burts, appointed Dr. A. M. Douglass as chairman of that committee, with power to name his four associates, but to date nothing has been heard from Dr. Douglass.

